# Three-dimensional kinematic motion analysis of a daily activity drinking from a glass: a pilot study

**DOI:** 10.1186/1743-0003-3-18

**Published:** 2006-08-16

**Authors:** Margit Alt Murphy, Katharina S Sunnerhagen, Bo Johnels, Carin Willén

**Affiliations:** 1 Dept. of Clinical Neuroscience – Rehabilitation Medicin, University of Göteborg, Sweden; 2 Dept. of Clinical Neurology, University of Göteborg, Sweden

## Abstract

**Background:**

Development of reliable and objective evaluation methods is required, particularly for natural and goal-oriented upper-extremity tasks. Three-dimensional imaging measurement techniques have turned out to be a powerful tool for a quantitative and qualitative assessment of multijoint movements. The purpose of this study was to develop and test a method of three-dimensional motion analysis for the activity "drinking from a glass" and describe the drinking task with kinematic variables in control subjects.

**Methods:**

A protocol was developed for the drinking activity including the set-up of cameras and positions of the markers and the subject. The drinking task included reaching, forward transport with glass, drinking, back transport and returning the hand to the initial position. An optoelectronic system was used for the three-dimensional kinematic motion capture. Movement times, velocities, joint angles and interjoint coordination for shoulder and elbow were computed and analyzed for twenty control subjects. Test-retest consistency was evaluated for six subjects.

**Results:**

The test protocol showed good consistency in test-retest. Phase definitions for the drinking task were defined and verified. Descriptive kinematic variables were obtained for movement times, positions, velocities and joint angles for shoulder and elbow joint. Interjoint coordination between shoulder and elbow joint in reaching phase showed a high correlation.

**Conclusion:**

This study provides a detailed description of the three-dimensional kinematic analysis of the drinking task. Our approach to investigate and analyze a goal-oriented daily activity has a great clinical potential. Consequently, the next step is to use and test this protocol on persons with impairments and disabilities from upper extremities.

## Background

The upper extremity has an important role in several daily activities such as eating, drinking, clothing, grooming, writing, as well as in different sports and leisure activities. These activities require coordination of multiple joints and involve both the musculoskeletal and neural systems [1]. Impairment of upper extremity is one of the most common sequels following CNS lesions [2,3] and is also frequent in patients with musculoskeletal impairments involving the upper extremity. A dysfunction in the upper extremity can significantly limit a person's level of activity and participation in their social and physical environment [4].

Upper extremity function is in neurological settings generally assessed with observer-initiated standardized measures based on ordinal scales, e.g. Fugl-Meyer Assesment, Frenchay Arm Test, Motor Assessment Scale, Action Research Arm Test, or as timed tests, e.g. Box and Block Test, Nine Hole Peg Test [5-7]. These outcome measures are reliable and sensitive for measuring gross changes in functional performance but have less sensitivity to smaller and more specific changes. Furthermore, despite the extensive experience in using these observer-initiated measures by clinicians, the subjectivity of these tests cannot be denied.

A better understanding of human movement requires more objective testing and accurate analysis of motion to describe the arm movements more precisely and specifically during functional testing. Kinematic analysis is one such method. Kinematics describes movements of the body through space and time, including linear and angular displacements, velocities and accelerations, but without reference to the forces involved [8,9]. Three-dimensional imaging measurement techniques, including optoelectronic systems, have turned out to be a powerful tool for a quantitative assessment of movement in all degrees of freedom. The models for lower extremity movements and gait analysis have been well established in biomechanical and clinical research and are now applied to the detailed diagnosis and treatment planning of patients. However, the variety, complexity and range of upper-extremity movements is a challenge to assessment and interpretation of data and the clinical routines for three-dimensional analysis in upper extremities are not fully established [10].

Movement analysis of reaching can provide precise quantitative and qualitative data of arm movement in space including movement velocities and accelerations. Joint angles and interjoint coordination can be calculated. In addition to the assessment of performance, kinematic measures can be useful for elucidating the motor strategies in goal-oriented tasks, as well as for evaluating upper-extremity therapies [7]. Most studies with kinematic analysis of upper extremities involve reaching movement carried out in the horizontal plane with the arm supported and in highly constrained conditions [7,11]. There are, however, an increasing number of studies with more "natural" reaching or pointing movements were in some cases even grasping an object is included [12-15].

Only a few studies have analyzed a functional task for upper extremity with three-dimensional kinematic analysis. Two of these studies have had the intention to attain data for upper limb kinematics in order to support the development of upper limb joint replacements [16,17]. Murgia et al investigated the motor control of wrist movements in two activities of daily living (jar opening and carton pouring) in four healthy persons [18]. One pilot study investigated the two-dimensional forearm movement during transport phase of drinking activity, with focus on effects of concentric and eccentric exercise training in elderly healthy women [19].

Kinematics studies have shown that reaching and grasping movements vary according to the goal and constraints of the task. For example, a pointing movement has different kinematics than a movement combined with grasping an object, in the same way that reaching movement kinematics are different depending on if the real-life object is present or not [9,20]. Studies of natural and goal-oriented movements are of particular relevance to clinical practice since they provide essential information of person's real capabilities [7,10].

There are no studies, in our knowledge, which have analyzed the whole drinking movement with kinematic characteristics. The starting-point for this study was to investigate and analyze this daily activity with kinematic analysis without physical restraints on the normal movement of drinking. We were also interested to explore the potential of this method for use in clinical practice. To get answers for these considerations it is necessary to develop a method of three-dimensional analysis of the drinking activity and gather the reference data for control persons. The aims of the present study were:

1. To develop a protocol and test the consistency of that protocol for the three-dimensional motion analysis of an daily activity "drinking from a glass",

2. To obtain descriptive group data for this drinking task in control subjects.

## Methods

### Subjects

The study group was based on a sample of convenience and included 20 control subjects (9 male and 11 female). The mean age was 48.2 years (range 31–64). The subject's height measurements were collected by self report. The length of the right arm was measured with a flexible measuring tape, arm adducted, inward rotated and elbow in 90 degrees flexion and defined as a distance between acromion and styloid process of ulna. The subjects mean height was 171.5 cm (range 157–187), and the mean right arm length was 61.1 (range 55–68). Inclusion criteria were: subjects with right dominant hand who were, in their own opinion, in "good health". Exclusion criteria were: the presence of musculosceletal or neurological problems that could affect the function of the arm. The study was approved by the Ethics Committee, Göteborg University, Sweden. All subjects received written information about the study and gave their consent before entering the study.

### Research set-up and procedure

A standardized test protocol was developed by testing a range of different marker positions, camera positions and subject positions. The final protocol met the measurement goals and did not interfere with or physically restrict the natural movement of drinking.

### The ProReflex motion capture system

The three-dimensional motion analysis was performed with a ProReflex Motion Capture System (Qualisys, Sweden). Data was transferred to Windows-based data acquisition software (Qualisys Track Manager). This system includes an advanced optoelectronic camera system that produces clean and accurate 3D data. The measurement accuracy is better at high frequencies (120 Hz–240 Hz) and is dependent on the size of markers. The system has been shown to calculate angles within 0.07 degrees of the actual angle [21]. Data analysis itself was performed with special software developed in MATLAB (The Mathworks, Inc).

Ballshaped markers, positioned on the body, reflect infrared light from camera flashes, and only those markers are displayed on the computer image. The markers image produces X, Y and Z coordinate values throughout the measured movement. The coordinate system was defined with X-axis directed forward (anteriorly), Y-axis directed laterally and Z-axis directed upward (superiorly).

**Figure 1:**
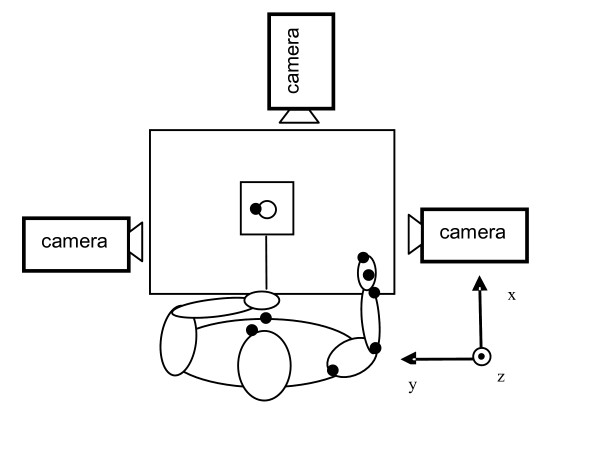
View from above of the set-up for the drinking activity with the XYZ coordinate system. The subject is represented with the arm in the initial position and marker sites are shown as black dots.

In the present study three cameras with a capture rate of 240 Hz were used. The cameras were positioned around the testing area as shown in Figure 1. The system was calibrated to a measurement volume of 75 × 75 × 65 cm and validated with a person sitting in the measurement area to ensure the visibility of markers throughout the drinking activity. The length of the camera capture period was set to 10 seconds, which was enough for a person to drink one swallow. A web camera was also used during measurements to complement motion data with synchronized video data.

### Marker sites

Nine spherical 12 mm and 19 mm reflective markers were attached to the skin with double-sided tape. The markers were positioned on the superficial bony prominences to reduce the effect of skin movement and to facilitate marker replacement in repeated testing. Similar marker positions have been used in other kinematics studies [11-13,21]. Markers were placed on the index finger (distal interphalangeal joint – DIP II), hand (third metacarpophalangeal joint – MCP III), wrist (styloid process of ulna), elbow (lateral epicondyle), shoulder (in the middle part of acromion), thorax (upper part of sternum), face (highest point on the left cheek) and two markers were placed on the object (near to the upper and lower edge of drinking glass). The thorax marker was used as a reference point to control amount of trunk displacement during the measurement.

### Set-up and procedure

All subjects performed the drinking movement with their right arm. Subjects were seated on a 43 cm high, strait-back chair in front of a 72 cm high table. A hard non-translucent plastic drinking container was used, since glass would reflect the camera flashes and disturb the motion capture. The drinking glass had a 7 cm diameter with a 9.5 cm height (volume 2.4 dl) and was filled with 1.5 dl water (half-full), and placed at a distance of 30 cm from the table edge, in a marked area 8 × 8 cm in the midline of the body. The set-up is shown in Figure 1.

In the start position, the subjects were sitting against the chair back, feet on the floor. Right arm was pronated with the hand resting on the table and wrist line close to the edge of the table. Subjects were asked to find a comfortable sitting position with right upper arm in vertical and adducted position and approximately 90 degrees flexion at elbow. The subject's left hand was resting on the lap.

Drinking movement included reaching and grasping the glass with all fingers (no fingers at the bottom), lifting the glass from the table and taking a drink (one swallow), placing the glass back on the table inside the marked area and returning to the start position. Subjects were instructed to sit against the chair back during the whole drinking task. This was also verified by a marker on the thorax, which provided exact kinematic displacements of the trunk during the whole task. The intention was to keep the drinking activity as normal as possible and let the subjects sit close enough to reach the drinking glass without their back leaving the chair support.

The subjects were allowed to try the drinking movement a few times to find a comfortable sitting position. When ready, the test leader announced, "you can start now", and the subject started the drinking task at a comfortable self-paced speed. Every subject was recorded for at least three and at most six trials in one testing session, depending on how well the computer could track the markers automatically.

To assess the consistency of the test protocol we performed test-retest on six randomly chosen subjects. Those six subjects were first tested according to the protocol. Then the subject left the measurement area and markers were removed. After a 5–10 minutes break the markers were replaced and subject was tested a second time.

### Data analysis and raw data handling

After the recording process, each of the markers were identified in Qualisys Track Manager and reviewed to ensure that the markers were tracked correctly throughout the data capture. In some recordings certain markers were partly hidden or merged with other markers and could not be tracked automatically. While, it was possible to perform a manual analysis of this data, this would demand an excessive amount of work and was considered not feasible according to the goal of this study. From all recordings (test-retest included), 7 % of the recordings were excluded due to high segmentation and gaps in data. In the final analysis the first three successful recordings from every subject were used and the mean of those were calculated as a final measurement value for each subject.

The data was transferred to the MATLAB software for further analysis. For every recording we calculated and plotted coordinate data showing position, velocity and acceleration. The drinking task was broken down in five logical phases: reaching, forward transport, drinking, back transport, returning. Phase definitions are described in detail under the results.

The goal was to find and define parameters that could render us clinically useful information and be comparable for different patient groups in later studies. After analyzing the graphical plots from the recordings the kinematic data analysis was focused on following variables:

• **Movement times **were calculated for the whole movement (total movement time) and separately for each phase based on phase definitions.

• **Peak velocities, **were determined for the different movement phases from tangential velocity traces of hand marker.

• **Time to peak velocity and percentage of time to peak velocity **were calculated for reaching phase.

• **Joint angles **were computed from the position data for elbow flexion/extension, for shoulder flexion/extension in sagittal plane and abduction/adduction in frontal plane. The elbow angle was determined by the angle between the vectors joining elbow and wrist markers and the elbow and shoulder markers. Shoulder angle was determined by the angle between the vectors joining the shoulder and elbow markers and the vertical vector from the shoulder marker toward the hip. Joint angles for different movement phases and the range for all movement were calculated.

• **Interjoint coordination **was calculated with correlation coefficient (Pearson product moment correlation) between the shoulder and elbow joint excursions for reaching phase from rawdata in Matlab software.

## Statistical analysis

Statistical analyses were performed with SPSS (Statistical Packages for Social Sciences, 11.0). Descriptive statistics including mean, standard deviation and 95% confidence intervals (CI) were calculated for the study group of 20 subjects and for test-retest data. The mean of the three recordings was used in statistical calculations.

The difference between test and retest was analyzed with a paired *t*-test with alpha level at 0.05 and with hypothesis testing based on confidence intervals of the test-retest data. The agreement between test and retest was evaluated with 95% limits of agreement (LOA) method [22,23]. The 95% LOA were calculated as the mean of difference ± 1.96 standard deviations of difference. This method calculates the limits within which expected differences between two measurements will lie with 95% probability. To check the assumptions of the limits of agreement the differences were plotted against the average of the two measurements for every variable.

## Results

### Phase definitions and movement times

The drinking task was broken down into five logical phases: reaching (includes grasping), forward transport with glass to mouth, drinking, back transport with glass (includes release of grasp) and returning the hand to the initial position. The mathematical and dynamical properties of kinematic data were used to determine the start and the end of each phase. Five subsequent phases for the drinking movement were defined and verified for all measurements (Table 1). Based on the phase definitions the movement times for each phase and for the whole movement (total movement time) were calculated and are displayed Table 2.

**Table 1:** Phase definitions for drinking task.

**Phase name**	**Start**	**Detected by**	**End**	**Detected by**
**Reaching (includes grasping)**	Hand movement begins	Hand marker velocity surpassed 2% of the peak velocity	Hand begins to move towards the mouth with the glass	Elbow angle is in maximal extension
**Forward transport (glass to mouth)**	Hand begins to move towards the mouth with the glass	Elbow angle is in maximal extension	Drinking begins	Hand marker velocity returned to (5 %) of the peak velocity
**Drinking**	Drinking begins	Hand marker velocity returned to (5 %) of the peak velocity	Drinking ends	Hand marker velocity surpassed 5% of the peak velocity
**Back transport (glass to table, includes release of grasp)**	Hand begins to move to put the glass back to table	Hand marker velocity surpassed 5% of the peak velocity	Hand releases the glass and begins to move back to initial position	Elbow angle is in maximal extension
**Returning (hand back to initial position)**	Hand begins to move back to initial position	Elbow angle is in maximal extension	Hand is back resting in initial position	Hand marker velocity returned to 2% of the peak velocity

**Table 2:** Kinematic variables for the control subjects (n = 20).

**Kinematic variables**	**Mean**	**SD**	**95% CI**
***Movement times (s)***			
Reaching	1.21	0.22	1.11–1.31
Forward transport	1.15	0.19	1.06–1.24
Drinking	1.71	0.44	1.51–1.92
Back transport	1.77	0.37	1.60–1.94
Returning	1.00	0.14	0.94–1.07
Total movement time	6.84	1.00	6.38–7.32
			
***Peak velocity (PV) (mm/s)***			
PV for reaching	551	78.3	514–587
PV for forward transport	273	50.4	249–296
PV for back transport	228	66.8	196–259
PV for returning	560	79.1	523–597
Time to PV in reaching (s)	0.41	0.10	0.37–0.46
Time to PV in reaching (%)	34.3	5.7	31.7–37.0
			
***Joint angles (°)***			
*Shoulder (sagittal plane)*			
Initial position	5.5	2.7	4.2–6.8
Grasping (maximal flexion)	48.9	5.1	46.5–51.2
Drinking (maximal flexion)	53.5	7.0	50.2–56.8
Range	48.3	7.5	44.8–51.8
			
*Shoulder (frontal plane)*			
Initial position	15.0	4.0	13.1–16.9
Reaching (abduction)	27.8	6.0	25.0–30.7
Grasping (maximal adduction)	10.6	4.6	8.5–12.8
Drinking (maximal abduction)	39.3	13.0	33.2–45.4
Range	28.7	10.5	23.8–33.6
			
*Elbow*			
Initial position	105.0	6.8	101.8–108.2
Grasping (maximal extension)	42.5	7.3	39.1–45.9
Drinking (maximal flexion)	136.4	3.8	134.6–138.1
Range	93.9	8.1	90.1–97.7

Abbreviations: SD – standard deviation, CI – confidence interval

### Marker position analysis

**Figure 2:**
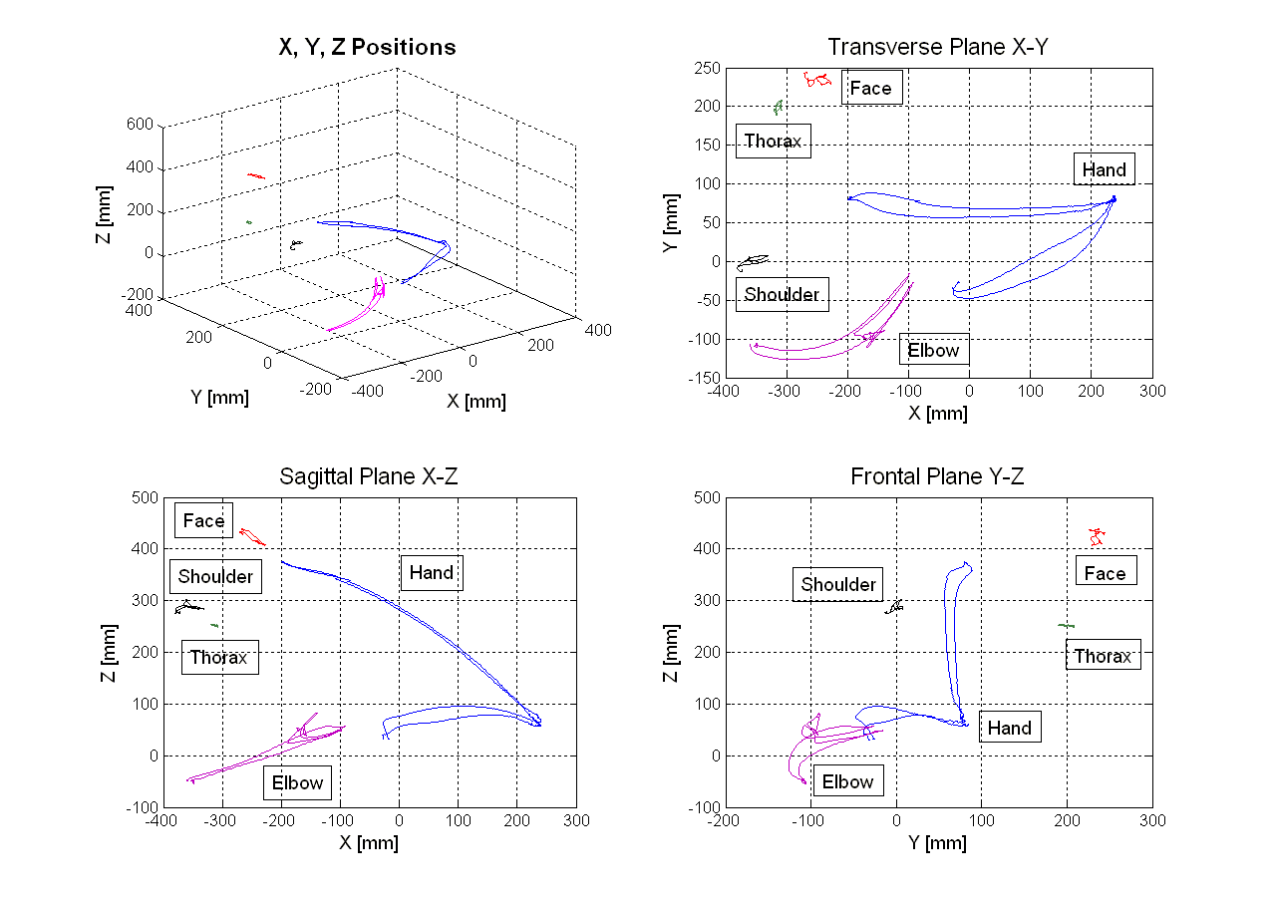
Position graphs with coordinate data from five markers, plotted in the three-dimensional graph and separately in transversal plane (X-Y), sagittal plane (X-Z), frontal plane (Y-Z) for one subject. X-axis directed anteriorly, Y-axix directed laterally, Z-axis directed upward.

Position graphs with coordinate data from each marker were plotted in three different dimensions (transverse, sagittal and frontal plane) for qualitative visual analysis. The position trajectories from five markers are shown in Figure 2.

Position graphs for all subjects were rather similar and had a characteristic path pattern. The elbow, wrist and hand markers had distinguished and smooth movement trajectories. The displacement data for the thorax, face and shoulder markers showed that the biggest displacement occurred in sagittal plane. The thorax marker remained relatively motionless with mean displacement 22.9 mm, confirming that subject's upper body was fairly still. Even face (mean displacement 47.6 mm) and shoulder markers (mean displacement 76.2 mm) had a small movement. The wrist, hand and finger markers had rather similar trajectories thus in all presented graphs only the hand marker is plotted representing the endpoint trajectory.

### Hand marker velocities

**Figure 3:**
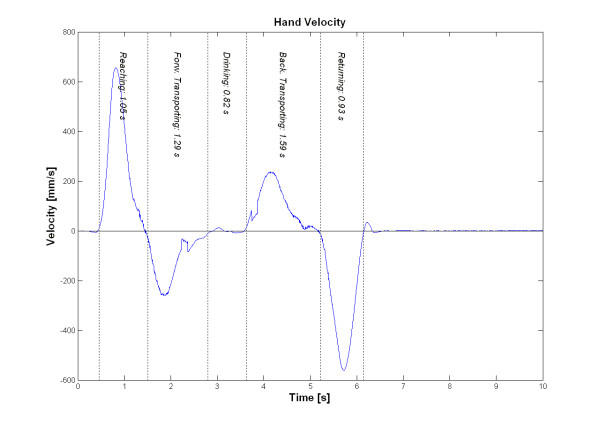
Hand marker velocity profile during the drinking task for one subject.

The tangential velocity profiles were calculated and plotted for the hand marker. Those velocity profiles were smooth and bell-shaped with one predominant peak. There were four distinctive velocity peaks for the whole movement task representing different movement phases. The velocity in the reaching and returning phases was approximately double the velocity in the transporting phases when the subject was holding the drinking glass. Hand tangential velocity decreased shortly before the glass was grasped or released. The back transport phase had a longer movement time and the peak velocity occurred earlier compared to the forward transport phase, thus indicating that placing the glass back on the table required more precision. The hand marker velocity profile for the drinking task is shown in Figure 3. Kinematic data for peak velocities in different phases, time to peak velocity and percentage of the peak velocity in reaching phase is shown in Table 2.

### Joint angles

**Figure 4:**
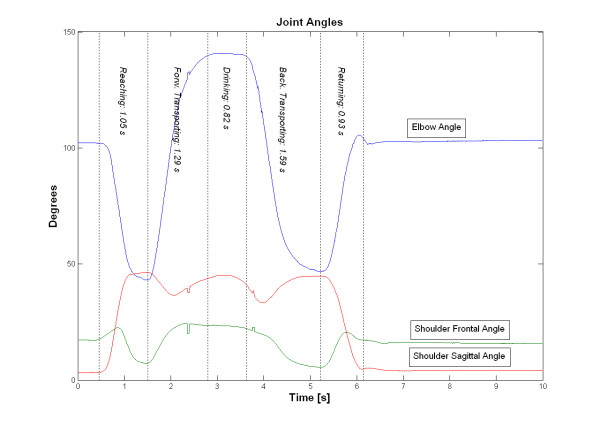
Joint angles for elbow (extension-downward, flexion-upward) and for shoulder in sagittal plane (flexion-up, extension-down) and in frontal plane (abduction-up, adduction-down).

Joint angles were calculated for the elbow (extension-flexion) and also for the shoulder in sagittal plane (flexion-extension) and in frontal plane (abduction and adduction). Joint angles versus timeline are displayed in Figure 4. Kinematic data for joint angles are presented in Table 2.

The elbow angle graph demonstrated a characteristic smooth movement pattern with the maximal elbow extension during grasping and releasing the glass. The maximal elbow flexion occurred during the drinking phase.

Shoulder flexion approached the maximal angle in the end of the reaching phase, and peaked shortly thereafter second time during the drinking phase. Shoulder abduction approach a small peak in the middle of reaching, indicating a slight half-circular arm movement in the reaching phase. The maximal adduction was achieved in the end of reaching phase and followed up by a maximal abduction during drinking phase.

The second part of the movement was almost identical with the first part in all graphs. The movement patterns were fairly similar for all subjects. Some stylistic differences were noticed with slightly different drinking styles. For example some subjects had their elbow near to the body (adducted) and others away from body (more abducted) in the drinking phase. For example the joint angle in abduction in drinking phase ranged from 20.9 to 74.9 degrees with a mean of 39.3 degrees and standard deviation 13.0 which was the highest standard deviation among the different joint angles.

**Figure 5:**
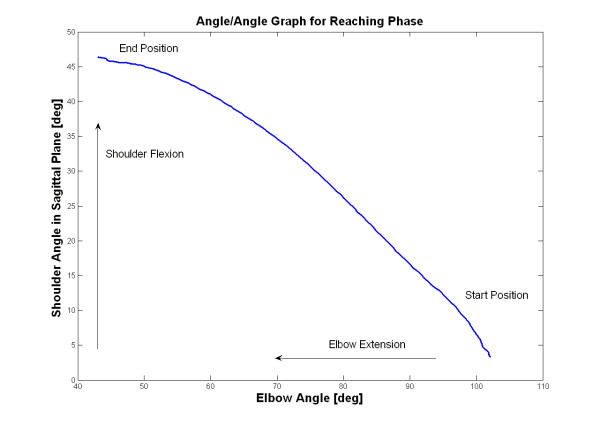
Interjoint coordination for the shoulder and elbow joint movements in reaching phase for one subject. Reaching movement starts from the right lower corner of the graph.

Interjoint coordination between the shoulder and elbow joint excursions during reaching phase showed a high correlation. Correlation coefficients (r) ranged from 0.92 to 0.98 with a mean value of 0.96 (SD 0.02). Interjoint coordination for the shoulder and elbow joints were plotted on an angle/angle graph for the reaching movement and are shown in Figure 5. Trajectory for interjoint coordination was smooth and continuous, forming an almost linear correlation between elbow and shoulder joint excursions.

### Test-retest consistency

The mean difference between test and retest, 95% confidence intervals of the difference, and 95% limits of agreement (LOA) were calculated for all kinematic variables. Statistics are presented in Table 3 for total movement time, peak velocity, and joint angels, representing the central components of kinematic analysis.

**Table 3:** Test-retest consistency for kinematic variables. The mean difference, 95% confidence intervals of mean difference and 95% limits of agreement in test and retest for total movement time, peak velocity and joint angles (n = 6).

**Kinematic variables**	**Mean difference (95%CI)**	**95% CI of mean difference**	**95% LOA**
Total movement time (s)	0.00	-0.22, 0.23	-0.43, 0.44
Peak velocity in reaching (mm/s)	-17.1	-52.2, 18.0	-84.0, 49.7
Shoulder abduction in drinking (°)	1.5	-2.4, 5.5	-5.98, 9.01
Shoulder flexion in drinking (°)	0.12	-1.4, 1.6	-2.79, 3.02
Elbow extension in grasping (°)	-0.16	-2.7, 2.4	-5.0, 4.7
Elbow flexion in drinking (°)	0.02	-1.7, 1.7	-3.3, 3.3

According to the assumptions of the limits of agreement, the differences did not vary in any systematic way over the range of measurement and all measurements were within the 95% limits of agreement. All values of mean difference were close to the zero and the widths of the 95% CI of difference and the 95% LOA were narrow. Hypothesis testing based on confidence intervals of test and retest data confirmed that the mean values of the retest were within the 95% confidence intervals for the first test. Based on these analyses we can say that there were confidently no differences between test and retest with 95% probability.

## Discussion

The present study provides a detailed three-dimensional kinematic analysis of the drinking task in control subjects. A standardized test protocol for the drinking task was developed, including the set-up of cameras, measurement volume, location of markers and position of the subjects. The test protocol demonstrated a good consistency in test-retest and provided clear and accurate results. The phase analysis which divided the drinking task into five sequential phases was unique for the present study. Kinematic data are presented for the movement times, marker positions, velocities and joint angles for the control subjects.

The drinking activity is a complex task in terms of kinematics. It contains several different movements as reaching, grasping, transporting the glass and drinking. In the present study, five sequential phases were identified: reaching, forward transport, drinking, back transport and returning. This phase analysis gives a logical and easy observable structure for the drinking task and provides the possibility to investigate different variables separately in each phase. There are no studies, to our knowledge, which have presented the phase definitions for the whole drinking movement; hence the phase analysis applied for the drinking task in this study is unique.

The concept of three-dimensional joint movements and joint angles is a quite unusual for clinicians. It is common to think of two-dimensional movement in different planes. To make this study more obtainable for clinicians and more comparable with other clinical studies, we divided the shoulder elevation into abduction and flexion and recalculated the values for elbow angle into an anatomical angle rather the technical/mathematical angle values. One limitation of this study, for example, is that we have not analyzed the rotations in shoulder and elbow joints or joint angles in the wrist joint. Wrist joint motion and forearm rotations could provide additional information of different drinking strategies. Although the system has the potential to provide this information, a much more complex set-up and analysis would be required. The goal of this study was not to measure the joint angles in all joints and in all degrees of freedom. Our intention was to collect informational and clinically useful data for the drinking task with the existing camera system and according to the current stage of software development.

We have used surface markers and computed the joint angles as the angles between the corresponding vectors joining the adjacent markers or vertical vector. It must be understood that those joint angles do not pass through the centers of rotation of the joints, thus they are not the true joint angles. However, placing the markers on the well-defined superficial bony prominences increases the reproducibility of data on different occasions. This was confirmed by the good consistency in test-retest in this study as well in other studies using surface markers [21].

Earlier studies of reaching movements have reported that trunk movement is acting both as stabilizer and as an integral component in positioning the hand close to the target [24]. Several studies have also shown that when reaching within arm's length, healthy subjects use minimal trunk displacement in contrast to hemiparetic subjects who use a compensatory strategy involving trunk recruitment [24-26]. In the present study the object was placed within an arm's length and the subject could reach the glass while sitting against the chair back during the whole task. Any unintentional trunk displacement was also measured using a marker on the thorax. The intention was to keep the drinking movement as natural as possible but allow some trunk displacement during the task.

Interjoint coordination for the elbow and shoulder joint excursions during a reaching movement is shown to be highly coupled in normal subjects, but has a significant decrease in stroke subjects with hemiparetic arm [11,12]. Levin et al suggested that a measure of interjoint coordination can give us clinically beneficial information about the subject's motor function. In the present study we found a high correlation between the shoulder and elbow joint excursions in reaching phase indicating a good interjoint coordination. Several studies have suggested using angle/angle graphs for qualitative analysis of interjoint coordination [11-13]. We have constructed the angle/angle graphs for the reaching movement and found a smooth and almost linear curve between elbow and shoulder joint excursions.

All measurements systems including the kinematic systems, suffer from measurement error. A typical average value for measurement error is estimated to be 2–3 mm in all dimensions in gait analysis [8]. Turner-Stokes et al found the absolute mean difference (differences between two measurements in either direction are treated as positive) in joint range to be 2.3° for shoulder and 2.7° for the elbow in repeated measures in the bowing arm of string-playing musicians when the whole measurement system was dismantled and re-set-up [21]. Replacing the markers produced slightly greater differences than repositioning and recalibration of the whole system [21]. In this study we tested the consistency regarding the replacing the markers and the repositioning the subject. The absolute mean difference in test-retest for shoulder abduction was 2.7°, for shoulder flexion 1.1°, for elbow extension 2.0° and for elbow flexion 1.2°. These values are comparable with results in study of Turner-Stokes et al.

The analysis of upper extremity tasks requires a measurement set-up and camera system which can track all the necessary markers throughout the whole movement. This has been a challenge in the present study. From total 112 recordings we had to exclude 8 recordings (7%), because of the high segmentation and gaps in data. This data lost is acceptable considering the biomechanical complexity in the upper extremity movement analysis [10]. The problem with segmentation and gaps in data have also been reported in other studies, especially when few (two to four) cameras are used and when the movement is complex in degrees of freedom [17,20,21]. As reported in other studies, the set-up of camera systems and data analysis in special software programs required a good collaboration between clinicians and engineers in the present study [10,21]. Results of this study corroborate that that the use of the existing motion capture system, as used in the presented protocol, still has some limitations and requires further refinement to be feasible for clinical use with persons with neurological disorders. The main clinical benefit of this study is the establishment of the phases of an important activity of daily living (ADL). The establishment of the phases can simplify the camera measurement system and allow for increased clinical use. In addition, the phases provide a descriptive methodology to classify strategies of drinking task of healthy persons.

## Conclusion

Kinematic analysis has great possibilities to be used as an outcome measure in clinical research especially when optoelectronic camera systems become more readily available in clinical settings. Our approach to investigate and analyze a goal-oriented daily task has a great clinical potential. However a certain degree of standardization is indispensable in order to obtain repeatable and reliable results. Further development of objective and reliable evaluation methods for upper extremity tasks is required, particularly for natural and goal-oriented movements. Consequently the next step is to use and test this protocol on persons with impairments and disabilities from upper extremities e.g. persons with neurological and musculoskeletal diseases.

## Authors' contributions

MAM contributed to the concept and design, acquisition, analysis and interpretation of data, drafting and completion of the manuscript. KSS contributed to conception and design, subsequent planning of study, analysis and interpretation of data, and revised the manuscript critically. BJ contributed to conception and design, subsequent planning of study, and revised the manuscript. CW contributed to conception and design, subsequent planning of study, analysis and interpretation of data, and revised the manuscript critically. All authors read and approved the manuscript to be published.
